# Defining predictors for successful mechanical ventilation weaning, using a data-mining process and artificial intelligence

**DOI:** 10.1038/s41598-023-47452-7

**Published:** 2023-11-22

**Authors:** Juliette Menguy, Kahaia De Longeaux, Laetitia Bodenes, Baptiste Hourmant, Erwan L’Her

**Affiliations:** 1 Medical Intensive Care Unit, CHRU de la Cavale Blanche, Bvd Tanguy-Prigent, 29609 Brest Cedex, France; 2 LATIM INSERM UMR 1101, Université de Bretagne Occidentale, 29200 Brest, France

**Keywords:** Physiology, Health care, Risk factors

## Abstract

Mechanical ventilation weaning within intensive care units (ICU) is a difficult process, while crucial when considering its impact on morbidity and mortality. Failed extubation and prolonged mechanical ventilation both carry a significant risk of adverse events. We aimed to determine predictive factors of extubation success using data-mining and artificial intelligence. A prospective physiological and biomedical signal data warehousing project. A 21-beds medical Intensive Care Unit of a University Hospital. Adult patients undergoing weaning from mechanical ventilation. Hemodynamic and respiratory parameters of mechanically ventilated patients were prospectively collected and combined with clinical outcome data. One hundred and eight patients were included, for 135 spontaneous breathing trials (SBT) allowing to identify physiological parameters either measured before or during the trial and considered as predictive for extubation success. The Early-Warning Score Oxygen (EWSO_2_) enables to discriminate patients deemed to succeed extubation, at 72-h and 7-days. Cut-off values for EWSO2 (AUC = 0.80; Se = 0.75; Sp = 0.76), mean arterial pressure and heart-rate variability parameters were determined. A predictive model for extubation success was established including body-mass index (BMI) on inclusion, occlusion pressure at 0,1 s. (P0.1) and heart-rate analysis parameters (LF/HF) both measured before SBT, and heart rate during SBT (global performance 62%; 83%). The data-mining process enabled to detect independent predictive factors for extubation success and to develop a dynamic predictive model using artificial intelligence. Such predictive tools may help clinicians to better discriminate patients deemed to succeed extubation and thus improve clinical performance.

## Introduction

Acute respiratory failure requiring mechanical ventilation (MV) is frequent within Intensive Care Unit (ICU) patients, and a large proportion of these patients will require MV for more than 24-h^1^. Although lifesaving, MV also causes numerous life-threatening complications. MV weaning and endotracheal extubation are a major challenge during the ICU stay and might be considered as soon as possible^2^. From the moment of endotracheal intubation, the clinician must consider two difficulties during the weaning decision process which are prolonging MV unnecessarily, or extubating the patient earlier, both associated with an inherent risk of increased morbidity and mortality^3, 4^. Indeed, the longer is the MV duration, the higher is the risk of baro-volotraumatic lesions, ventilator-associated pneumonia (VAP) or other infectious complications. Although fast discontinuation of MV is the primary goal, premature extubation is also associated with weaning failure and complications^4, 5^.

In some reports, weaning accounts for more than 40% of the total ventilation period. If 60% patients may experiment a simple weaning process of less than 24-h, weaning may be more difficult for the remaining 40% patients^6, 7^. If various strategies such as active weaning either using a once-daily trial of spontaneous breathing (SBT), pressure-support ventilation, or automated weaning has proven to reduce the weaning duration and the inherent MV complication rate, such a process remains too long and/or still uncertain in terms of outcome^8–12^. In addition, no difference has been shown in favour of one method over another in terms of both weaning test technique and weaning test duration^13, 14^. The protocolization of mechanical ventilation weaning is essentially that allows its application and success^15, 16^.

Indeed, in order to identify the best time for secure extubation, weaning should be considered by daily screening for objective clinical improvement criteria (pre-test probability of weaning). Several predictive factors of endotracheal reintubation have been assessed in previous studies (rapid-shallow breathing index [RR/Vt], MV duration, cough strength)^17–20^. However, all these indicators depicted insufficient sensitivity/specificity and other indicators are mandatory to improve our clinical decision robustness. While the discriminative capacity of a 30-min SBT seems important to predict extubation success, failure of endotracheal extubation still occurs (15 to 20%)^21–24^. Causes for MV weaning failure are complex, multifactorial and not only related to the patient’s respiratory status^25–27^.

Weaning from mechanical ventilation can be divided in 3 steps. The first is to look for objective criteria for weaning. The second is to perform a weaning test, and extubation is the last step.

Physiologically, heart rate and blood pressure vary during the respiratory cycle, corresponding to the activity of the autonomic nervous system (ANS).

Mechanical ventilation disturbs the intra-thoracic pressure regimes resulting from heart–lung interactions and thus their regulation^28, 29^. The influence of the ANS contributes to the respiratory sinus arrhythmia process and heart-rate variability (HRV)^30, 31^. Several studies have depicted significant HRV variations in patients under MV, especially while considering the weaning process, in an attempt to predict clinical evolution^32–35^. Transposed to mechanical ventilation weaning situations, the evaluation of these warning scores to predict the success of extubation and thus, limit the complications of prolonged mechanical ventilation or extubation failure might be of interest^36^.

The main objective of this project was to identify parameters associated with a successful weaning. The second objective was to create a predictive model for weaning success either during the pre-test and the SBT phases, while using artificial intelligence and machine-learning.

## Material and methods

### Study design

Data analysis was performed on the ReaSTOC database, which is an ongoing prospective physiological and biomedical signal data warehousing project including all consecutive patients admitted to our adult medical ICU (ClinicalTrials.gov identifier NCT02893462). A previous publication has described the design and conduct of the ReaSTOC study^37^. The protocol #29BRC18.0080 was approved on November the 5th 2019 by the local ethics committee of our institution (*Comité d’Ethique du CHRU de Brest IRB #2018CE.27).* Written informed consent was waived according to French legislation (Law n°2012–300 March 5th 2012 also called “Loi Jardé” https://www.legifrance.gouv.fr/eli/jo/2012/3/6), in accordance with the ethical standards of our local human experimentation review board and with the Helsinki Declaration of 1975.

### Population

All patients included within the database and under MV for more than 24 h were considered eligible for weaning, according to a standardized protocol. A patient could be analyzed for several SBT during his ICU stay, if he had not experienced prior extubation; thus, only the first period of invasive MV was considered if endotracheal intubation was deemed necessary after an extubation failure.

Exclusion criteria were pregnancy, discontinuation of treatment (terminal extubation), participation decline by the patient or relatives, time of MV < 24 h, self-extubation, patients under legal protection or without social security, or patients with missing cardiac variability data.

Patients were classified in three groups according to their ventilation weaning difficulty, as proposed by the 6th international consensus conference^3^.

Group 1 – “simple weaning” included patients who successfully completed the first weaning test, followed immediately by extubation. In the literature, this group represents 69% of weaned patients and the prognosis is rather favorable with a 5% ICU mortality and a 12% in-hospital mortality.

Group 2 – “difficult weaning” included patients who achieved one to three SBT before extubation, within less than 7 days after the first attempt.

Group 3 – “prolonged weaning” included patients requiring more than 3 SBT, or less than 3 tests but within more than 7 days after the first attempt, before extubation. Within groups 2 and 3, ICU mortality is equal or higher than 25%^6, 27^.

### Weaning protocol

According to a standardized protocol, established for years within our ICU^16^, the ability of a patient to perform a SBT (Pre-Test) was assessed daily by the nursing team while considering the above criteria: no sedation, no or low dose of inotropic or vasopressor treatment, adapted response to simple orders, FiO_2_ < 50% and PEEP ≤ 5cmH_2_O. If these conditions are met, the SBT is initiated by a ventilator disconnection and the use of a T-piece, for a maximal 30 min duration, using an oxygen flow rate adjusted to a predetermined SpO_2_ target.

Failure of the SBT was defined by signs of poor clinical tolerance including: respiratory rate (RR) > 35 cycles/min, SpO2 < 90%, change of > 20% in heart rate (HR) or blood pressure, sweating, agitation or consciousness disorders. If any of these conditions occurred, SBT was immediately stopped and the patient was re-placed under mechanical ventilation. If none of these conditions were observed and if the patient had an effective cough, the SBT was considered successful and the patient was extubated.

Successful MV weaning was defined as extubation after a successful SBT, without reintubation within 72 h.

### Data collection

Demographic and physiological parameters, medical condition prior to ICU admission, length-of-stay and MV, as well as hemodynamic and respiratory data before, during and after the weaning test were collected for each patient. At the end of the weaning test, the decision whether or not to extubate was recorded and justified in the case of non-extubation or reintubation. Decisions to limit treatment or terminally extubate were specified and documented.

Weight balance was assessed at the time of SBT and considered as either negative (decrease in body weight since admission), neutral (same body weight as admission), or positive (increase in body weight since admission).

Body-Mass Index (BMI) was assessed on admission according to the actual patient’s height and weight. It was subsequently divided into 5 classes: low-weight-denutrition (BMI < 18.5 kg/m^2^), normal (BMI = 18.5–24.9 kg/m^2^), overweight (BMI = 25–29.9 kg/m^2^), obesity (BMI = 30–39.9 kg/m^2^), or morbid obesity (BMI > 40 kg/m^2^).

Continuous photoplethysmography (PPG) data were recorded 30-min prior to SBT, during SBT, and 30-min after extubation, at a 75 Hz frequency (Phillips Intellivue MP70 monitor) via the SYNaPSE extraction software (System for Nonintrusive Physiological Signal Exploration LTSI INSERM UMR 1099). Recording of PPG curves was used to perform HRV analysis, in the temporal (RMSSD, triangular index), frequency (VLF, LF, HF, LF/HF), and non-linear domains (SD1, SD2, SD2/SD1, Approximate—Sample and Shanon entropies). Such approach using plethysmogram has been validated within a prior study^37, 38^.

The respiratory parameter called Early-Warning Score Oxygen (EWSO_2_) was defined in the observational study by Viglino et al.^39^ and its variations during the weaning test were collected.

### Statistical analysis

In the absence of an a priori hypothesis, no number of subjects was calculated and all available data were used for analysis. Results are presented as mean and standard deviation, unless specified otherwise. The comparison of quantitative variables in each group was performed using Student or Wilcoxon tests according to distribution’s normality, and qualitative variables were compared using Khi-2 or Fischer tests. Outcome independent predictors were identified by logistic regression; performance of these predictors for a specific cut-off value was determined while calculating the area under the receiving operating characteristics curve (AUC for ROC). Youden's J statistic (also called Youden's index) is a single statistic that captures the performance of a dichotomous diagnostic test. Its value ranges from −1 through 1 (inclusive), and has a zero value when a diagnostic test gives the same proportion of positive results for groups with and without the disease, i.e. the test is useless. A value of 1 indicates that there are no false positives or false negatives, i.e. the test is perfect.

Probability of an event for MV weaning was determined using Kaplan–Meier analysis.

All analysis were performed using R +  + (v1.5.03; Zebrys, Toulouse, France). A p-value equal or less than 0.05 was considered statistically significant.

Predictive models were developed by artificial intelligence and machine learning using the ZGPD model (TADA, MyDataModels, Biot, France), that uses evolutionary and genetic algorithms and symbolic regression. A model was obtained by search for associations between the variables among 40% of the iterations performed on the database. These associations of variables defining the model were subsequently tested on 30% of the data, and finally validated on the remaining 30% of the data.

The selection of the most relevant models among all those created was based on the global performance score of the model and its relevance to clinical practice. The global performance score reflects the strength of the model and is based on three statistical components: accuracy (ACC), Matthews correlation coefficient (MCC) and the area under the curve (AUC).

## Results

**Figure 1:**
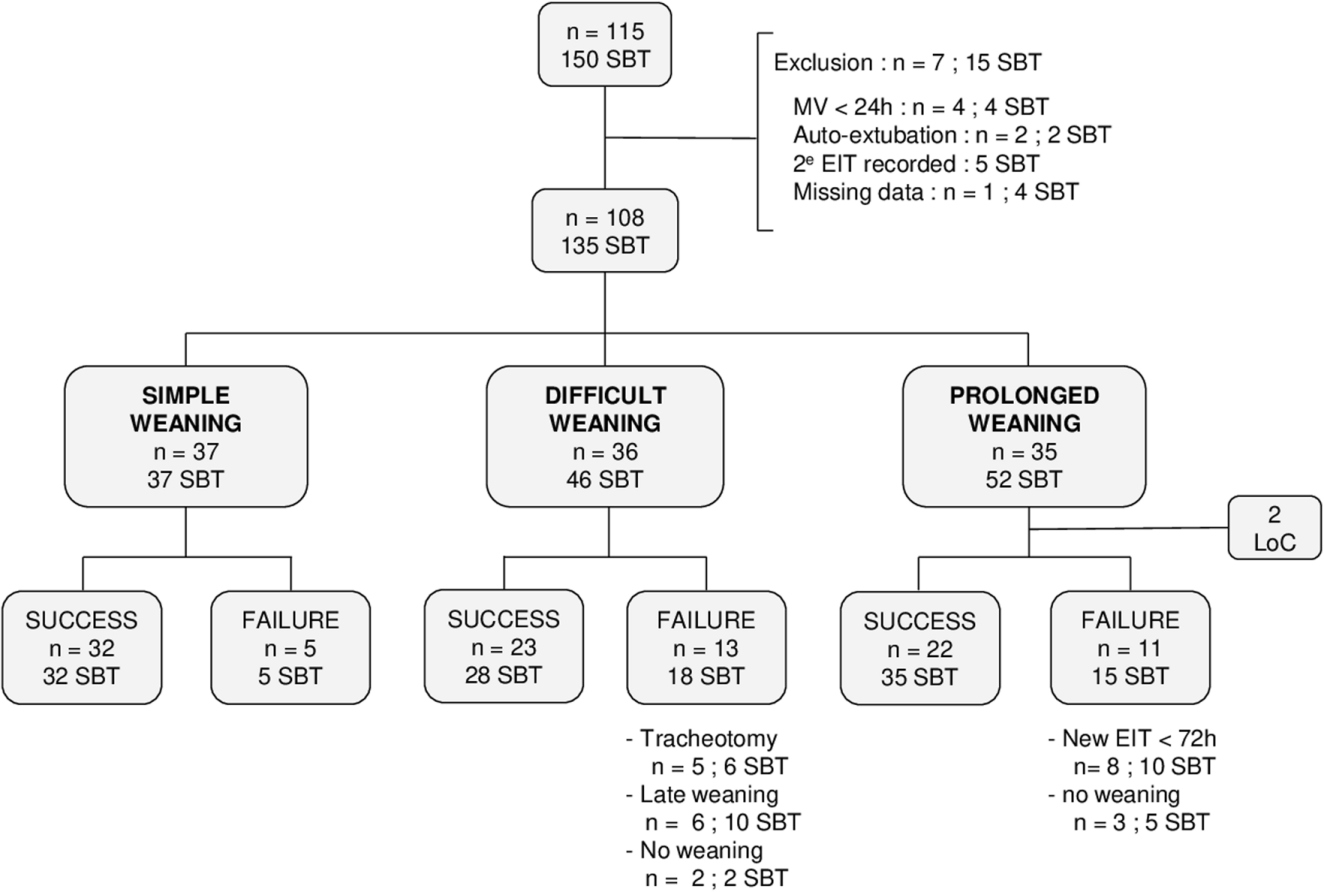
Patients’ flow-chart. SBT: Spontaneous Breathing Test; MV: Mechanical Ventilation; EIT: Endotracheal Intubation; LOC: Lost of Contact; Success: Successful SBT and no reintubation within 72-h; Failure: failure of the SBT, or successful SBT but reintubation within 72-h.

The data from 108 patients were analysed, representing a total of 135 SBT recordings (Fig. 1). Patients’ physiological characteristics are reported within Table 1, and groups are compared according to the extubation status. No significant difference was depicted in between the 2 groups, except for body weight balance (p = 0.0007) and immunosuppression status (p = 0.007).

**Table 1:** Patients’ physiological characteristics.

Extubation After SBT	YES 67 (49.63%)	NO 68 (50.37%)	Overall 135 (100%)	p value
Physiological Characteristics				
Sex Ratio (F/M)	25/42 (37.3%)	17/51 (25.0%)	42/93 (31.1%)	0.17
Age	62.9 (12.1)	62.4 (11.4)	62.7 (11.7)	0.80
BMI	26.4 (6.3)	27.4 (6.1)	26.3 (6.3)	0.88
SAPS 2	54.9 (16.4)	53.4 (17.0)	54.2 (16.7)	0.60
SOFA	3.37 (2.17)	3.79 (2.22)	3.59 (2.20)	
Weight Balance				**0.0007**
Negative	13 (19.7%)	9 (13.4%)	22 (16.54%)	–
Neutral	20 (30.3%)	5 (7.46%)	25 (18.80%)	–
Positive	33 (50.0%)	53 (79.1%)	86 (64.66%)	–
BMI Class				0.49
Low Weight—Denutrition	5 (7.7%)	4 (6.1%)	6 (6.9%)	–
Normal	25 (38.5%)	20 (30.3%)	45 (34.4%)	–
Overweight	20 (30.8%)	25 (37.9%)	45 (34.4%)	–
Obesity	11 (16.9%)	16 (24.2%)	27 (20.6%)	–
Morbid Obesity	4 (6.2%)	1 (1.5%)	5 (3.8%)	–
Medical History				
Cardiac Disease	38 (56.7%)	38 (55.9%)	76 (56.3%)	1
Respiratory Disease	38 (56.7%)	33 (48.5%)	71 (52.6%)	0.44
Immunosuppression	2 (3.0%)	13 (19.1%)	15 (11.2%)	**0.007**
Smoking	19 (46.3%)	18 (40%)	37 (43.0%)	0.82
Alcohol Abuse	21 (61.8%)	23 (56.1%)	44 (59.7%)	0.34
Medical Condition				
Current Pathology				0.16
Respiratory	25 (37.3%)	33 (48.5%)	58 (42.96%)	–
Hemodynamic	9 (13.4%)	10 (14.7%)	19 (14.07%)	–
Neurologic	25 (37.3%)	15 (22.1%)	40 (29.63%)	–
Cardiac Arrest	7 (10.4%)	5 (7.35%)	12 (8.89%)	–
Other	1 (1.49%)	5 (7.35%)	6 (4.44%)	–
Hemodynamic Unstability	2 (3.0%)	3 (4.4%)	5 (3.7%)	1
Atrial Fibrillation	10 (14.9%)	16 (23.5%)	26 (19.3%)	0.29
Arterial Hypertension	14 (20.9%)	22 (32.4%)	36 (26.7%)	0.19
Diuretics	9 (13.4%)	11 (16.2%)	20 (14.8%)	0.84
Vasopressors	56 (83.6%)	57 (83.8%)	113 (83.7%)	1
Beta-blockers	6 (%)	10 (%)	16 (%)	0.24

Results are presented as number (%). A p value equal or below 0.05 was considered statically significant and depicted in black tone.

F: female; M: male; SBT: Spontaneous Breathing Test; BMI: Body-Mass Index; SAPS 2: Simplified Acute Physiologic Scale; SOFA: Sequential Organ Failure.

Significant values are in bold.

Table 2 reports the clinical and outcome parameters in between groups, while considering the extubation status. The causes for SBT failure were cardiovascular (68.4%), respiratory (28.1%), neurological (1.8%) and other (1.8%). The different interfaces used after extubation were either room air, O_2_ via nasal canula, high flow nasal oxygen (HFNO), or non-invasive ventilation (NIV). Oxygenation parameters evaluation before and during SBT highlights a lower EWSO_2_, SpO_2_ values and higher FiO_2_, RR and O_2_ flow rate during SBT (p < 0.01 for parameters assessed prior to SBT and p < 0.001 for parameters assessed during SBT). Outcomes were also different in between groups, according to the extubation status.

**Table 2:** Clinical and outcome parameters.

Extubation After SBT	YES 67 (49.63%)	NO 68 (50.37%)	Overall 135 (100%)	p value
Respiratory parameters				
Before SBT				
FiO_2_ (%)	26 (6)	30 (8)	28 (7)	**0.002**
PEEP (cm H_2_O)	5.2 (1.0)	5.2 (1.3)	5.2 (1.1)	0.99
VT (mL/kg)	7.5 (0.7)	7.5 (0.7)	7.5 (0.7)	0.19
P0.1 (cm H_2_O)	1.7 (1.0)	2.2 (1.6)	2.0 (1.4)	0.11
Respiratory Rate (b/min)	24 (6)	26 (6)	25 (6)	**0.02**
SpO_2_ (%)	95 (3)	94 (3)	95 (3)	**0.002**
Heart Rate (b/min)	89 (19)	95 (20)	92 (20)	0.07
SAP (mmHg)	133 (24)	133 (22)	133 (23)	0.94
MAP (mmHg)	88 (14)	87 (14)	88 (14)	0.47
EWSO_2_	17.9 (6.9)	14.1 (5.8)	16.0 (6.6)	**0.005**
SBT number	2 (3)	2 (2)	2 (2)	0.79
During SBT				
O_2_ flow (L/min)	2.6 (1.4)	3.9 (1.9)	3.2 (1.8)	** < 0.0001**
Heart Rate (b/min)	91 (17)	101 (21)	96 (20)	**0.008**
Respiratory Rate (b/min)	26 (7)	33 (8)	29 (8)	** < 0.0001**
SpO_2_ (%)	95 (3)	91 (5)	93 (5)	** < 0.0001**
SAP (mmHg)	140 (27)	145 (26)	142 (27)	0.25
MAP (mmHg)	93 (15)	95 (17)	94 (16)	0.25
EWSO_2_	14.8 (5.3)	9.9 (4.4)	12.4 (5.4)	** < 0.0001**
After SBT				
O_2_ flow (L/min)	4.1 (9.5)	NA	NA	NA
Heart Rate (b/min)	94 (21)	NA	NA	NA
Respiratory Rate (b/min)	25 (8)	NA	NA	NA
SpO_2_ (%)	95 (3)	NA	NA	NA
SAP (mmHg)	138 (23)	NA	NA	NA
MAP (mmHg)	91 (16)	NA	NA	NA
EWSO_2_	9.8 (9.5)	NA	NA	NA
Outcomes				
Overall MV duration (days)	7.0 (8.6)	14.2 (13.3)	10.5 (11.7)	**0.0004**
VFD in ICU (days)	2.8 (3.2)	3.4 (3.7)	3.1 (3.5)	0.29
VFD at Day28 (days)	21.6 (9.3)	14.6 (10.8)	18.1 (10.7)	**0.0001**
ICU LOS (days)	13.5 (12.4)	24.3 (16.9)	18.8 (15.7)	** < 0.0001**
Hs LOS (days)	38.5 (41.8)	39.8 (35.4)	39.1 (38.6)	0.85
Reintubation ≤ 72h (n)	14 (20.9%)	19 (31.1%)	33 (25.8%)	0.26
Reintubation ≤ Day7 (n)	14 (20.9%)	18 (29.5%)	32 (25.0%)	0.36
Never Weaned (n)	0 (0%)	8 (12.1%)	8 (6.0%)	**0.002**

Results are presented as number (%). A p value equal or below 0.05 was considered statically significant and depicted in black tone.

SBT: Spontaneous Breathing Test; FiO_2_: inspiratory oxygen fraction; PEEP: positive end expiratory pressure; VT: volume tidal; P0.1: negative pressure measured 100 ms after the initiation of an inspiratory effort performed against a closed respiratory circuit; SpO2: pulse oximetry; SAP: systolic arterial pressure; MAP: mean arterial pressure; NIV: noninvasive ventilation; MV: mechanical ventilation; VFD: ventilatory free day; LOS: length-of-stay; Hs: hospital; NA: not applicable.

Significant values are in bold.

Table 3 depicts the spontaneous breathing tests outcome, reasons for failure which were mainly related to a post-extubation cardiovascular failure and mechanical ventilation weaning characteristics and strategies.

**Table 3:** Spontaneous breathing test outcome and weaning characteristics.

Extubation After SBT	YES 67 (49.6%)	NO 68 (50.4%	Overall 135 (100%)	p value
Reason for SBT Failure				
Cardiovascular	–	39 (68.4%)	–	NA
Respiratory	–	16 (28.1%)	–	NA
Neurologic	–	1 (1.75%)	–	NA
Other	–	1 (1.75%)	–	NA
Interface after Extubation				
AA	14 (21.2%)	–	–	NA
O2	36 (54.5%)	–	–	NA
HFNC	1 (1.75%)	–	–	NA
NIV	15 (22.7%)	–	–	NA
Weaning Classification				** < 0.0001**
Easy	37 (55.2%)	0 (0%)	37 (27.2%)	–
Prolonged	13 (19.4%)	39 (57.4%)	52 (38.2%)	–
Difficult	17 (25.4%)	29 (42.6%)	47 (34.6%)	–

Results are presented as number (%). A p value equal or below 0.05 was considered statistically significant and depicted in black tone.

SBT: spontaneous breathing test; AA: ambient air; HFNC: high flow nasal canula; NIV: noninvasive ventilation; NA: not applicable.

Significant values are in bold.

Table 4 depicts logistic regression used to identify independent parameters associated with extubation, after SBT, and extubation success at 72-h and Day-7. Parameters are presented with their best cut-off value, determined by ROC curves analysis.

**Table 4:** Independent outcome predictors identified by logistic regression.

	P	Cut-off	AUC	Youden’s Index	Se	Sp	PPV	NPV
Extubation after SBT								
Weight Balance (kg)	0.01	0.5	0.64	0.28	0.79	0.49	0.61	0.70
SD1 before SBT (ms)	0.03	25.8	0.57	0.18	0.49	0.69	0.62	0.56
SpO_2_ during SBT (%)	**0.04**	91.5	**0.76**	0.41	0.56	0.85	0.78	0.67
EWSO_2_ before SBT	0.001	12.8	0.67	0.28	0.47	0.81	0.71	0.60
EWSO_2_ during SBT	**0.006**	11.1	**0.80**	0.50	0.75	0.76	0.75	0.76
Extubation Success 72-h								
BMI	< 0.0001	23.6	0.67	0.33	0.61	0.73	0.44	0.84
P0.1 before SBT (cmH_2_O)	0.02	3.3	0.53	0.28	0.30	0.98	0.88	0.73
SpO_2_ during SBT (%)	0.007	95.5	0.61	0.24	0.87	0.24	0.31	0.89
MAP during SBT (mmHg)	**0.02**	104.5	**0.72**	0.35	0.52	0.83	0.50	0.84
SAP during SBT (mmHg)	0.02	145.5	0.68	0.33	0.69	0.64	0.38	0.86
EWSO_2_ during SBT	0.03	11.9	0.61	0.23	0.74	0.49	0.33	0.85
SD2 prior SBT (ms)	0.01	19.9	0.58	0.22	0.82	0.41	0.33	0.86
Estimated FiO_2_ after extubation (%)	0.03	29.0	0.60	0.27	0.54	0.74	0.33	0.87
EWSO_2_ after extubation	** < 0.0001**	16.2	**0.72**	0.43	0.91	0.52	0.29	0.96
Extubation Success Day-7								
BMI (kg/m^2^)	< 0.0001	23.6	0.67	0.36	0.63	0.73	0.44	0.85
MAP before SBT (mmHg)	< 0.0001	93.5	0.68	0.29	0.56	0.73	0.42	0.83
SAP before SBT (mmHg)	< 0.0001	142.5	0.63	0.33	0.59	0.74	0.44	0.84
EWSO_2_ before SBT	< 0.0001	22.3	0.54	0.17	0.94	0.23	0.29	0.92
EWSO_2_ during SBT	< 0.0001	12.4	0.61	0.22	0.77	0.45	0.31	0.86
EWSO_2_ after extubation	** < 0.0001**	16.2	**0.72**	0.43	0.91	0.52	0.29	0.96
LF before SBT (Hz)	0.03	120.0	0.57	0.18	0.78	0.40	0.31	0.84

A p value equal or below 0.05 was considered statistically significant. Parameters with an AUC higher than 0.70 are depicted in black tone.

Cut-off: best value for the parameter, determined by ROC Curve analysis; ROC: Receiver Operating Characteristic; AUC: Area Under the Curve; Se: Sensibility; Sp: Specificity; PPV: Positive Predictive Value; NPV: Negative Predictive Value; SBT: Spontaneous Breathing Test; SD1: Poincaré plot standard deviation perpendicular the line of identity; SD2: Poincaré plot standard deviation along the line of identity; LF: peak frequency of the low-frequency band of the plethysmography signal (0.04–015 Hz); SpO2: pulse oximetry; EWSO2: early-warning score oxygen; BMI: body-mass index; P0.1: negative pressure measured 100 ms after the initiation of an inspiratory effort performed against a closed respiratory circuit; SAP: systolic arterial pressure; MAP: mean arterial pressure; estimated FiO_2_: estimated oxygen inspiratory fraction, derived from the mean oxygen flow rate.

Significant values are in bold.

Table 5 depicts ROC curves analysis enables to detect HRV parameters that may predict the probability for a never-weaned patient (SD2/SD1, Sample Entropy, Shanon Entropy).

**Table 5:** ROC curves characteristics for heart rate variability parameters and selected physiological characteristics in never-weaned patients.

	SD2/SD1 during SBT	SampEn during SBT	ShanEn during SBT	BMI admission	P0.1 before SBT	HR during SBT
AUC	0.74 [0.50, 0.97]	0.74 [0.50, 0.99]	0.74 [0.52, 1.0]	0.52 [0.32, 0.73]	0.74 [0.39, 1.0]	0.57 [0.37, 0.77]
Youden’s J	0.52	0.59	0.50	0.20	0.63	0.24
Sensibility	0.86 [0.06,0.87]	0.86 [0.03,0.94]	0.71 [0.07,0.96]	0.44 [0.25, 0.84]	0.80 [0, 1.0]	0.3 [0.48, 0.99]
Specificity	0.66 [0.43, 1.0]	0.73 [0.14, 1.0]	0.79 [0.29, 1.0]	0.75 [0, 0.7]	0.84 [0.4, 1.0]	0.94 [0, 0.6]
Cut-off	1.50	1.63	2.96	22.7	2.5	69.5
PPV	0.14 [0.11, 0.67]	0.17 [0.12, 0.64]	0.18 [0.11, 0.60]	0.12 [0.07, 0.21]	0.27 [0.16, 0.6]	0.30 [0.10, 0.58]
NPV	0.99 [0.96, 1.0]	0.99 [0.96, 1.0]	0.98 [0.96, 1.0]	0.95 [0.94, 1.0]	0.98 [0.95, 1.0]	0.94 [0.93, 1.0]

AUC: area under the curve; PPV: positive predictive value; NPV: negative predictive value; SBT: spontaneous breathing test; SD1: Poincaré plot standard deviation perpendicular the line of identity; SD2: Poincaré plot standard deviation along the line of identity; SampEn: sample entropy; ShanEn: Shanon entropy: Youden’s J: statistic value for performance of dichotomous diagnostic test: Cut-off: best value for the parameter, determined by ROC curve analysis.

**Figure 2:**
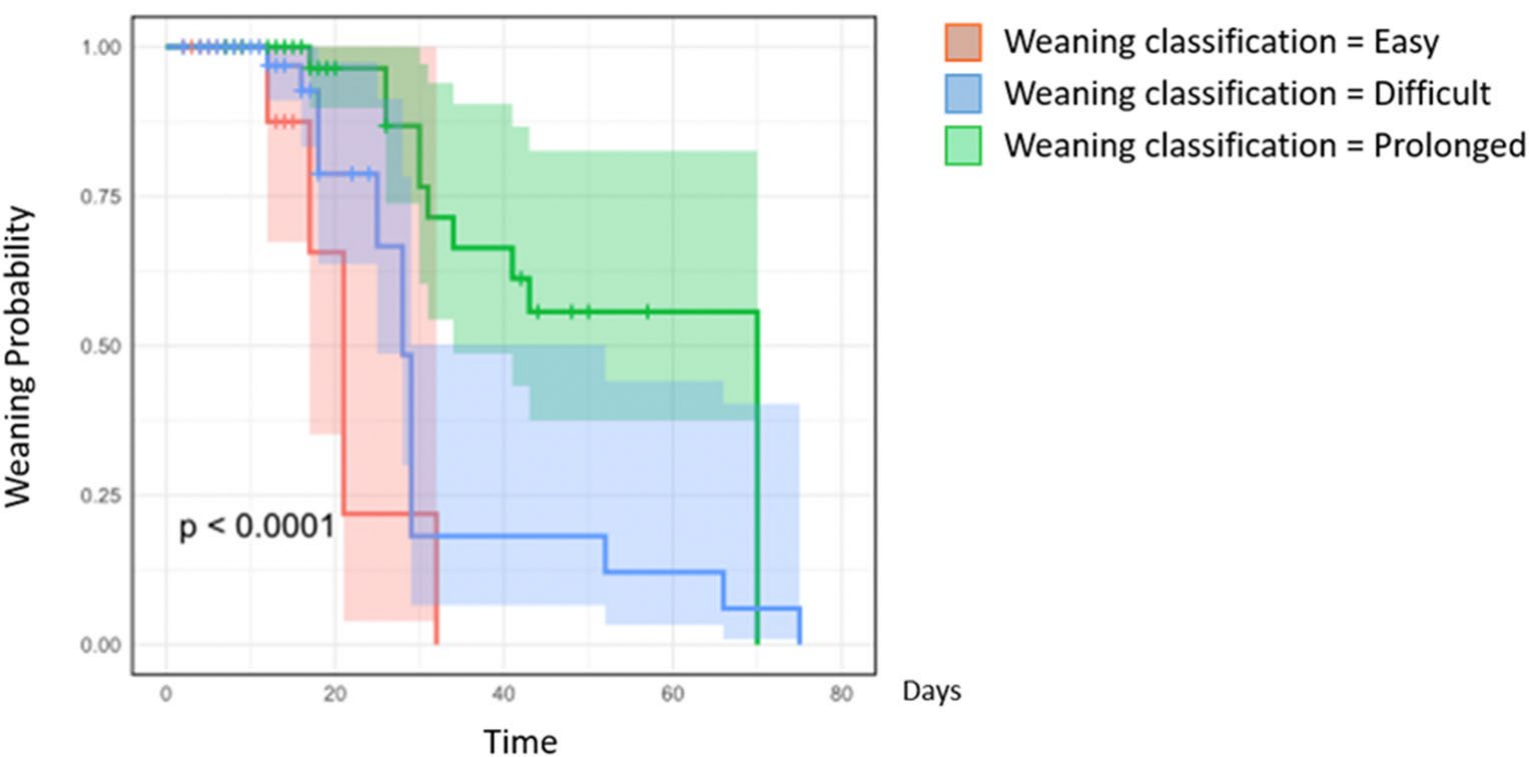
Kaplan–Meier Curve for weaning Probability according to the classification group. Weaning classification in 3 groups according to the Consensus definition clearly depicts weaning outcome^3^.

A Kaplan Meier curve was designed, demonstrating the probability of extubation failure in the 72 h according to the ventilation weaning group (Fig. 2). It confirmed that the probability of reintubation was lower in the simple weaning group and significantly more riskyr for prolonged and difficult weaning groups, all the more as the length of stay increases.

While using machine-learning, the best model to predicting extubation success at 72-h (no reintubation during the 3-days following SBT success and extubation) was composed of BMI on inclusion, P0.1 measured before the SBT, LF/HF before the SBT, and HR during the SBT (Global performance 70%; Accuracy 83%).

[https://viewer.mydatamodels.com/?modelId=9cb2556b-3b9a-49f4-adfa-2cb0690e367e].

This model is accessible in the “live predict” thumb index of the URL. Prediction can be obtained online while providing the values for the different parameters.

## Discussion

The current work demonstrated that in a medical ICU patient population, the outcome of mechanical ventilation weaning was associated with the duration of mechanical ventilation, ICU and global hospital length-of-stay. The data-mining process enabled to detect independent hemodynamic and respiratory predictive factors for extubation success and to develop a dynamic predictive model using artificial intelligence. These results may suggest that a rather simple model assessing physiological status prior to weaning and clinical response to a SBT might predict with a good level of confidence the overall weaning outcome. The evaluation of HRV was also confirmed as a valuable tool for weaning prediction, when combined with other respiratory parameters.

Choosing the appropriate time for weaning a patient under MV is crucial in order to reduce risks related to either prolonged ventilator support and to avoid premature weaning^3, 40,41^. Rapid-Shallow Breathing Index (RSBI) and the outcome of a SBT based on respiratory parameters are the most commonly used methods^3^, even if various RSBI thresholds and sensibilities have been argued in various population^42^. Until now, no single appropriate predictor, especially those only focused on respiratory parameters can be used to accurately predict weaning outcome^43^.

While several authors did use artificial intelligence to develop prediction models^44^, few authors did integrate HRV analysis despite promising results either in adults and premature infants and the fact that it may reflect either heart–lung interaction and respiratory command^45,46^. In a pilot study, the team from Barcelona did recently promote the combination of HRV analysis to traditional respiratory parameters measured to improve weaning readiness^32^. The model that best predicts outcome at day-3 either combines physiological characteristics (BMI on admission), respiratory drive (P0.1) and HRV status (LF/HF) that may be considered as indicators for a severity evaluation of the pathological process and a very simple clinical evaluation of the response to SBT (HR). One could note that the most frequent cause for SBT failure within our database was considered to be related to a cardiovascular failure, which therefore make sense to monitor a simple cardiovascular function tolerance parameter such as the heart rate during the test. Most of these indicators can be assessed prior to the T-tube trial on a stabilized patient, which may thus secure either the SBT period and the subsequent endotracheal extubation period. In case of severity detection with the model, either delaying extubation, or a systematic use of prophylactic measures (HFNC or NIV) might be proposed if SBT was considered a success.

The interest of the present work relies on its originality and methodology. To the best of our knowledge, no similar study using a prospective data-warehousing project has ever been performed in the ICU environment. Using such an approach enables to collect numerous parameters without any a priori, and the final analysis of so many parameters is made possible by artificial intelligence algorithms. Moreover, the creation of dynamic predictive models by machine learning is also innovative, while after validation of the derivation process such models may subsequently be used easily in clinical routine, at the patients’ bedside on any laptop, or even smartphones. It may thus enable to promote an optimized and more personalized approach for MV weaning.

Principal component analysis and neural networking are some other popular machine-learning methods that enable to explore connexion and correlation in between various parameters types that cannot be assessed while using conventional statistical approaches. The machine-learning methods that were used herein, while aiming to achieve similar goals, are slightly different while they promote genetic and evolutionary algorithms, that as compared to the previous methods are intrinsically compact, mathematically simplified, uses less power and also enable the use of small sample sizes, thus making it valuable for healthcare and clinical bedside evaluations.

Another methodological question that raises from the results section is the difference between the final AI model and the independent predictors detected using logistic regression. Such difference is explained by the fact that evolutionary algorithms uses multiple iterations over the dataset (from at least 100 to 1000 iterations), each time changing the reference population, thus constructing a more important population of candidate solutions and possibly explaining final differences in between predictors. However, while initially constructing models, all independent predictors defined by logistic regression were combined with other parameters considered of interest from a clinical point of view.

The clinical application of these various models is actually under process within our ICU to validate their usability in a daily life situation as well as to evaluate their interest in terms of clinical practice and prognosis improvement. Additional parameters such as non-invasive tidal volume evaluation using a time-of flight camera will be analysed, thus combining RSBI calculation to other parameters.

Our study may have several limitations. The first one is the monocentric characteristic of our medical ICU population. A more diversified population will be mandatory, prior to generalization of the algorithms. The second one is the accessibility and availability of HRV monitoring within most ICUs. Despite the robustness and the generic calculation of these parameters, they are not yet available in routine practice, while raw data are accessible within most medical datafiles but fewly exploited. Moreover, prediction dynamic models such as the ones developed herein may be considered as useful at the bedside while they integrate multiple and continuous changes in the patients’ clinical status overtime^47^, even if they cannot entirely eliminate the deviation caused by external factors, nor then reduce the risk of applying false prediction to an individual level. A third limitation of this work is that the type of oxygen therapy support used during SBT and after the extubation period was not studied. Furthermore, while we are routinely using T-tube SBT, the RSBI was not routinely available for our patients.

## Conclusion

If weaning from MV is a process well standardized within the general ICU population, uncertainty remains for several patients that will fail extubation. Various parameters have been studied within literature to predict extubation outcome. In this prospective datamining study, we were able to develop comprehensive dynamic models combining respiratory parameters to systemic ones. Further clinical studies will be mandatory to validate any clinical impact of such models on a clinical routine.

## Data Availability

All data and material related to the manuscript may be accessible from the corresponding author on reasonable request.

## Abbreviations

ACC: Accuracy
ANS: Autonomic nervous system
AppEn: Approximate entropy
AUC: Area under the curve
BMI: Body mass index
EWSO2: Early warning score oxygen
HFNO: High flow nasal oxygen
HF: High frequency
HR: Heart rate
HRV: Heart rate variability
ICU: Intensive care unit
LF: Low frequency
LF/HF: Low frequency/high frequency
MAP: Mean arterial pressure
MCC: Matthews coefficient correlation
MV: Mechanical ventilation
NIV: Non-invasive ventilation
P0,1: Occlusion pressure at 0,1 s.
PPG: Photoplethysmography
RMSSD: Root mean square of successive differences
ROC: Receiver operating characteristic
RR: Respiratory rate
SampEn: Sample entropy
SBT: Spontaneous breathing test
ShanEn: Shanon entropy
VAP: Ventilation associated pneumonia
VLF: Very low frequency
